# Runaway uncoupling in 2,4-dinitrophenol poisoning: Clinical and mitochondrial observations from two cases

**DOI:** 10.1016/j.toxrep.2025.102183

**Published:** 2025-12-08

**Authors:** Erik Lindeman, Tine Sommer, Märta Leffler, Shusuke Sekine, Eskil Elmér, Fredrik Sjövall, Wilhelm Wallquist

**Affiliations:** a Swedish Poisons Information Center, Solna Strandväg 21, Stockholm 171 54, Sweden; b Department of Intensive and Perioperative Medicine, Skåne University Hospital, Inga Marie Nilssons gata 47, Malmö 214 28, Sweden; c Mitochondrial Medicine, Department of Clinical Sciences Lund, Lund University, Lund, Sweden; d Department of Anesthesiology, Tokyo Medical University, Japan; e Clinical Neurophysiology, Medical Imaging and Physiology, Skåne University Hospital, Lund, Sweden

**Keywords:** DNP, Hyperthermia, Mitochondrial uncoupling, Postmortem redistribution

## Abstract

2,4-Dinitrophenol (DNP) is a potent mitochondrial uncoupler briefly marketed in the 1930s as a weight-reducing agent before being banned by the FDA after reports of severe toxicity. Since the early 2000s, DNP has reemerged as an illicit “fat-burner”, causing characteristic metabolic disturbances with a high risk of fatal outcome. We describe two Swedish cases of DNP poisoning: one fatal after suicidal ingestion and one non-fatal after use for weight reduction. Clinical data, mitochondrial respirometry, and analysis of gas exchange and ventilatory dynamics were used to characterize the metabolic disturbances under intensive care. The fatal case progressed within hours to respiratory acidosis, hyperthermia, severe hyperkalemia, and peri-mortem rigidity consistent with catastrophic ATP depletion. The non-fatal case showed similar but reversible toxicity, with sustained yet manageable hypermetabolism lasting more than a week. Serial platelet respirometry demonstrated a marked initial increase in uncoupled respiration, followed by a progressive decline with a functional half-life of 4.9 days. Together, these cases suggest a self-amplifying feedback loop central to DNP toxicity, in which excessive CO₂ production from mitochondrial uncoupling causes local acidosis that enhances mitochondrial DNP uptake. Glucose supplementation and hyperkalemia management are essential supportive measures, whereas active cooling and high minute ventilation may blunt this self-reinforcing metabolic acceleration. Severe poisoning may result in a state of “runaway uncoupling,” a term we propose for the catastrophic progression to death observed in numerous DNP poisonings. This feedback loop illustrates the unpredictable toxicokinetics of DNP and reinforces the FDA’s early conclusion: DNP is “unfit for human consumption”.

## 1 Introduction

2,4-Dinitrophenol (DNP) was first synthesized in the 19th century and briefly used as a food dye. This application was discontinued after studies demonstrated that the compound elevated body temperature, stimulated respiration and accelerated the onset of rigor mortis in laboratory animals [1]. DNP entered large-scale industrial use in France during World War I in the manufacture of munitions. Factory workers exposed to airborne DNP-dust and through skin contact, experienced weight loss, profuse sweating and hyperthermia, with several fatalities reported [1], [2].

In 1933, researchers at Stanford University reported that DNP induced weight loss by stimulating a hypermetabolic state [3]. This publication directly triggered the compound’s widespread use in the United States as an over-the-counter weight-reduction agent [4], [5]. However, by the late 1930s, numerous adverse outcomes had been reported, including skin changes, neuropathy and the development of so-called “dinitro-cataracts,” a condition that caused blindness in thousands [1], [4], [5], [6], [7]. Several cases of severe acute illness and death, apparently driven by uncontrolled hypermetabolism and often accompanied by profound hyperthermia, had also been reported [4], [8], [9]. In response, the U.S. Food and Drug Administration banned DNP in 1938, declaring it “extremely dangerous and not fit for human consumption” [1], [10]].

The mechanism underlying DNP-induced hypermetabolism was not fully elucidated until decades later. In 1948, Loomis and Lipmann provided the first mechanistic clue by demonstrating that DNP disrupted the coupling between “phosphate bond generation”—now recognized as ATP synthesis—and “oxidative reactions,” referring to cellular oxygen consumption [11]. DNP is a weak acid with a pKa of approximately 4, such that a small fraction remains undissociated at physiological pH [12]. In this form, DNP is lipophilic and readily crosses cellular and mitochondrial membranes, accumulating in the mitochondrial matrix (the most alkaline compartment of the cell) where it donates a proton. The resulting anionic form, stabilized by a delocalized π-electron system, possesses an unusual ability for a charged species – it can recross biological membranes. This enables DNP to function as a protonophore (Greek for “proton carrier”), a property shared by other weak organic acid uncouplers such as salicylate. Through continuous shuttling of protons into the mitochondrial matrix, DNP dissipates the proton gradient that normally drives ATP synthesis, leading to excessive heat production and compensatory hypermetabolism [13].

**Fig. 1:**
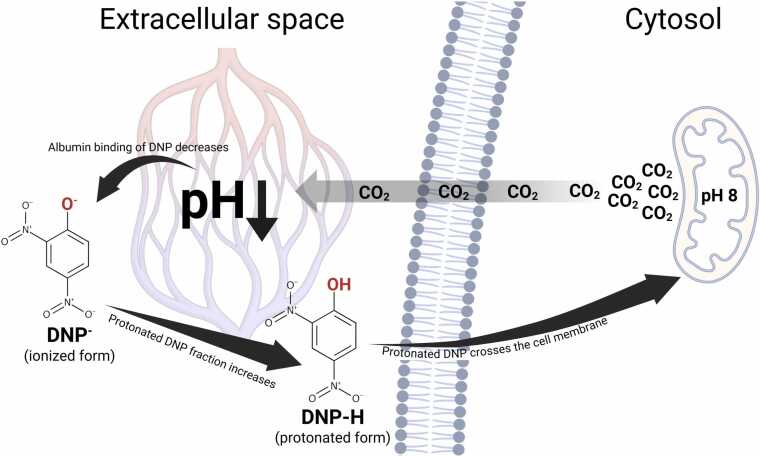
Proposed pH-dependent reinforcement mechanism in DNP toxicity. Excess CO₂ generated by hypermetabolism secondary to toxic uncoupling diffuses from mitochondria into the cytosol and extracellular space, where hydration of CO₂ lowers local pH. Acidosis has two major effects on DNP distribution: it reduces albumin binding, increasing the free fraction, and it shifts the acid–base equilibrium toward the protonated form (DNP-H). DNP has a pKa of 4.1, and according to the Henderson–Hasselbalch relationship, a fall in extracellular pH to around 7.0 would increase the protonated fraction (DNP-H) by a factor of 2–3. The protonated species is highly membrane-permeable and readily enters the alkaline mitochondrial matrix (pH ≈ 8), where it dissociates back to DNP⁻. Although DNP⁻ is an anion, its charge is extensively delocalized over an aromatic π-electron system, allowing it to cross the inner mitochondrial membrane and continue the protonophore cycle. Created in BioRender. Lindeman, E. (2025) https://BioRender.com/3gao4fq.

Following its 1938 ban, DNP-related poisonings became rare, apart from a local outbreak in Texas during the 1980s [1], [7], [10], [12]. Since the early 2000s, however, the compound has re-emerged through online sales as an illicit “fat-burner,” causing recurrent clusters of severe and often fatal intoxications, particularly among bodybuilders [10], [14], [15]. In Sweden, nine forensically confirmed DNP-related deaths have occurred since 2010, and the Swedish Poisons Information Centre (PC) receives an average of 5–6 suspected cases each year [16]. Here, we describe two recent Swedish cases of DNP toxicity in bodybuilders, managed in close consultation with the Swedish PC. Drawing on these cases and prior reports, we propose that DNP toxicity may be self-reinforcing through a feedback loop in which hypermetabolism promotes mitochondrial DNP uptake, further intensifying uncoupling. A schematic overview of the pH-dependent reinforcement mechanism is shown in Fig. 1. This hypothesis has important therapeutic implications, discussed below.

## 2 Case reports

### 2.1 Case 1

A young adult male was brought to the emergency department approximately one hour after ingesting twenty-five 200 mg capsules of 2,4-dinitrophenol (DNP) (total 5 g) together with 60 mg of clonazepam. He told ambulance personnel that he had taken a few DNP capsules the previous evening to “prime” his metabolism before the large ingestion, which was taken with suicidal intent.

On arrival he was alert and oriented. Body temperature was 37.4 °C, blood pressure 120/100 mmHg, heart rate 108 beats per minute, and respiratory rate 18 breaths per minute. A peripheral venous blood gas was normal: pH 7.40, partial pressure of carbon dioxide (pCO₂) 6.1 kPa, lactate 0.7 mmol/L, and glucose 5.1 mmol/L. One hour later he became restless and began sweating profusely. No temperature was recorded at that time. Because of escalating agitation, he was intubated 3.5 h after ingestion using rapid-sequence induction with propofol and rocuronium.

Shortly after intubation, the end-tidal carbon dioxide (EtCO₂) measured 17 kPa, and an arterial blood gas (ABG) obtained minutes later showed marked hypercapnia, with arterial pCO₂ (aB-pCO₂) 14 kPa and pH 7.10. Base excess and lactate remained within normal limits (0 and 1.5 mmol/L, respectively). Serum potassium was 5.0 mmol/L at this time. Almost immediately the patient developed bradycardia followed by asystole. Cardiopulmonary resuscitation (CPR) was initiated and continued for 1.5 h, after which he was pronounced dead. During CPR he developed peri-mortem rigidity, with flexed arms and markedly elevated airway pressures that necessitated manual bag ventilation. A single dose of dantrolene (2 mg/kg) was given but had no apparent effect. Core temperature rose to 40 °C, pCO₂ increased further to 25 kPa, and serum potassium rose to 11 mmol/L. Lactate could not be measured during resuscitation because of analyzer error.

DNP in blood was subsequently determined during forensic autopsy (see 3).

### 2.2 Case 2

A 32-year-old man was admitted to the intensive care unit after four days of using DNP for weight loss. His history included hypertension, proteinuria under nephrology follow-up, active anabolic-steroid use, and previous cocaine abuse. Two years earlier he had been hospitalized for adverse effects from DNP with a benign course. He had completed his first full day on a “target dose” of DNP 200 mg three times daily, as instructed by an online vendor, following a short dose-escalation phase. His cumulative intake over four days was 1.4 g. Approximately six hours after his final dose, he presented with anxiety, dyspnea, and a sensation of overheating.

On arrival he was agitated, diaphoretic, tachypneic (40 /min), tachycardic (126 bpm), and hypertensive (185/101 mmHg). Tympanic temperature was 36.8 °C. Because of worsening agitation, he was intubated and sedated within two hours of admission. An ABG before intubation showed respiratory alkalosis: pH 7.49, aB-pCO₂ 3.7 kPa, lactate 1.0 mmol/L, potassium 5.1 mmol/L.

**Fig. 2:**
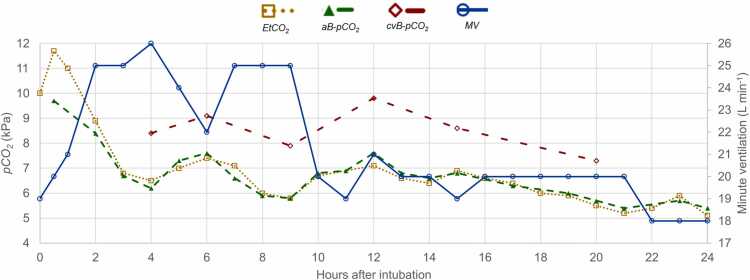
Minute ventilation (MV, right axis) and partial pressures of carbon dioxide (pCO₂, left axis) over time in Case 2. Arterial (aB-pCO₂) and end-tidal (EtCO₂) values correlated closely, while central-venous (cvB-pCO₂) values remained approximately 2 kPa higher than aB-pCO*₂*, consistent with increased CO₂ production from hypermetabolism induced by mitochondrial uncoupling.

A Foley catheter with a temperature probe placed after intubation showed a core temperature of 38.0 °C. He was ventilated in pressure-controlled mode with positive end-expiratory pressure (PEEP) 8 cm H₂O and driving pressure 15 cm H₂O, producing a minute ventilation (MV) of 18.6 L/min. Under these settings, aB-pCO₂ was 9.7 kPa and EtCO₂ 11.7 kPa. Increasing MV to 25.6 L/min lowered aB-pCO₂ to 7 kPa. During the first 24 h, both aB-pCO₂ and EtCO*₂* remained between 6 and 7 kPa despite MV > 20 L/min (Fig. 2). Central venous pCO₂ was consistently about 2 kPa higher than arterial values, while central venous oxygen saturation remained low at 60 % despite supranormal arterial oxygenation, hemodynamic stability, and normal lactate—findings consistent with hypermetabolism and excess CO₂ production.

Core temperature rose to 38.9 °C one hour after intubation. External ice packs were applied and a cooling catheter (Zoll Solex 7) inserted. Active intravascular cooling was initiated using a Thermoguard XP system (Zoll Medical Corporation, Chelmsford, MA, USA) at maximum output (144 W), reducing core temperature to < 38 °C within hours.

During the first hour after intubation, serum potassium rose from 5.0 to 6.7 mmol/L, prompting initiation of continuous renal replacement therapy (CRRT). At the same time, hypoglycemia (2.7 mmol/L) developed and was treated with an infusion of 50 % glucose (up to 75 g h⁻¹) to maintain euglycemia. The patient did not develop muscle rigidity; lactate remained normal, and body temperature, pCO₂, blood glucose, and potassium were subsequently controlled with these interventions.

A single 180 mg dose of dantrolene (≈ 1.6 mg/kg) was administered. Continuous rocuronium infusion was maintained to reduce muscle activity and suppress shivering during cooling.

**Fig. 3:**
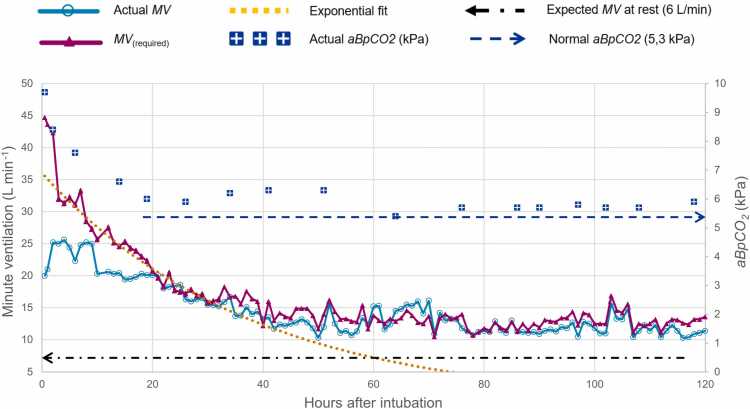
Ventilatory demand analysis in Case 2. Actual minute ventilation (MV, light blue) from ventilator log data is plotted alongside the calculated ventilation required to achieve normocapnia (MV_(required)_, purple), estimated using the measured EtCO₂–MV data and extrapolated to the level predicted to yield an end-tidal pCO₂ of 5.3 kPa (see Methods). The decline in MV_(required)_ between 2 and 32 h was well described by an exponential fit (yellow dotted line, *R²* = 0.96), corresponding to an apparent half-life of 26 h. Beyond 32 h, MV_(required)_ remained persistently elevated above the expected resting ventilation (6 L/min), while arterial (aB-pCO₂*,* blue squares) stayed supranormal.

From day 2 the patient remained hemodynamically stable, though signs of sustained hypermetabolism persisted. Hyperventilation with MV 12–15 L/min was required to maintain normocapnia for several days (Fig. 3). The intravenous glucose infusion continued until day 5, after which normoglycemia was maintained with enteral feeding.

Extubation was deferred until day 7 because of persistent delirium. On the day of extubation, a MV of 12 L/min was still needed to keep EtCO₂ at 5.3 kPa, indicating a persistently elevated resting metabolic demand. The patient ultimately recovered fully.

Blood for DNP analysis was drawn on day 1 (admission), and additional samples for mitochondrial-respirometry studies were obtained on days 1, 5, and 8. Ventilator-derived calorimetry was performed on day 6 (see 3).

## 3 Additional studies

### 3.1 DNP blood concentrations

In Case 1, a post-mortem whole-blood DNP concentration of 8.4 mg/L was determined in femoral blood taken at autopsy five days after death. In Case 2, blood concentrations measured 28 mg/L and 17 mg/L at 14- and 26-hours post-ingestion, respectively. Assuming first-order kinetics, this corresponds to an estimated half-life of 16.7 h. All analyses were performed at an accredited reference laboratory using liquid chromatography–tandem mass spectrometry (LC–MS/MS). Results were not available to guide clinical decision-making.

### 3.2 Mitochondrial respirometry

Mitochondrial function was assessed in Case 2 using high-resolution respirometry in platelets. Samples were collected on days 1 (admission), 5, and 8. Platelets were isolated by differential centrifugation from EDTA-anticoagulated whole blood and resuspended in autologous plasma [17]. Mitochondrial respiration was measured at 37 °C in MiR05 buffer using an Oxygraph-2k system (Oroboros Instruments, Innsbruck, Austria). After establishing stable Routine (basal) respiration, oligomycin (1 µg/mL) was added to inhibit ATP synthase, allowing quantification of oligomycin-insensitive (uncoupled) respiration (Leak). Maximal electron-transfer-system (ETS or ET) capacity was then determined by titration with FCCP (carbonyl cyanide-p-trifluoromethoxyphenylhydrazone). Finally, rotenone and antimycin A were added to block complexes I and III, and residual non-mitochondrial oxygen consumption (Rox) was subtracted from all values.

**Fig. 4:**
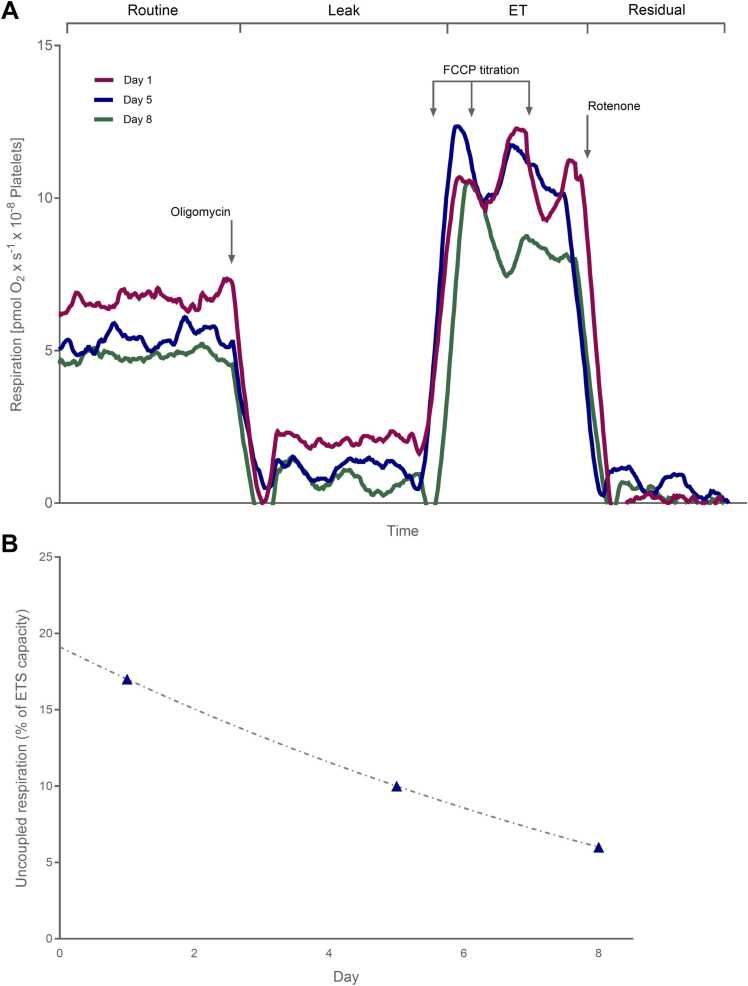
Decline in platelet uncoupled respiration over time in Case 2. (A) Oxygraph traces depicting mitochondrial respiration in platelets using a standard coupling-control protocol with consecutive additions of oligomycin (Leak respiration), FCCP titration (ET capacity), and rotenone/antimycin A (residual oxygen consumption). Uncoupled respiration was markedly elevated on day 1 and declined progressively over subsequent measurements. (B) Uncoupled respiration expressed as a percentage of maximal electron-transport-system (ETS) capacity on days 1, 5, and 8 (16.7 %, 10.4 %, and 6.3 %, respectively). An exponential decay fit (R² = 0.994) was consistent with a first-order process and enabled calculation of a functional half-life of approximately 4.9 days.

Uncoupled mitochondrial respiration in platelets was initially markedly increased and declined progressively over time. The uncoupling-control ratio (UCR), the fraction of Routine cellular respiration attributable to uncoupling, was 30.3 % on day 1, 23.7 % on day 5, and 13.5 % on day 8. Expressed relative to maximal ETS capacity, uncoupled respiration measured 16.7 % on day 1, 10.4 % on day 5, and 6.3 % on day 8. In historical control samples analyzed in the same laboratory, uncoupled respiration typically accounts for 1.5–2 % of ETS capacity. Functional measurements thus indicated an apparent half-life of 4.9 days, assuming exponential decay. The exponential fit was strong (R² = 0.994), supporting a first-order process (Fig. 4). Functional half-life may be affected by platelet turnover and may differ between tissues, points that are further developed in the Discussion. Platelets collected on days 5 and 8 and incubated with day-1 plasma exhibited a similar degree of uncoupling as freshly collected day-1 platelets, indicating that residual DNP in day-1 plasma was a major contributor to the early mitochondrial dysfunction. By contrast, plasma from days 5 and 8 did not induce uncoupling in DNP-naïve platelets (data not shown).

### 3.3 Ventilatory-demand analysis

Ventilatory demand was assessed by calculating the minute ventilation (MV₍_required_₎) required to maintain normocapnia (target pCO₂ = 5.3 kPa), based on logged EtCO₂ and actual MV values. The calculation was derived from the alveolar ventilation equation and adapted for clinical use by Wexler and Lok [18]: *MV*₍_required_₎ = *MV*₍_actual_₎ × (*EtCO₂*₍_actual_₎ / 5.3). EtCO₂ values closely paralleled aB-pCO₂ measurements (Fig. 2).

Peak calculated MV₍_required_₎ was 43.6 L/min, with a mean of 30.9 L/min during the first 24 h. The actual peak and mean ventilator settings were 25.6 and 22.1 L/min, respectively. Corresponding mean EtCO₂ and aB-pCO₂ values were 7.49 kPa and 6.83 kPa. After an initial stabilization period, the decline in MV₍_required_₎ between 2 and 32 h was well described by an exponential fit (R² = 0.96), corresponding to an apparent half-life of approximately 26 h. Beyond 32 h, MV₍_required_₎ showed no consistent decay but fluctuated noisily around a persistently elevated plateau, while arterial pCO₂ remained supranormal (Fig. 3).

### 3.4 Ventilator-derived calorimetry

Resting energy expenditure was assessed in Case 2 on day 6 using ventilator-derived calorimetry. Carbon dioxide production (VCO₂) was measured with a Servo-U ventilator (Getinge, Sweden). Energy expenditure was calculated using the formula proposed by Stapel *et al.* [19]: *Energy expenditure (kcal day⁻¹) = 8.19 × VCO₂ (mL/min)*. While still sedated and mechanically ventilated, the patient’s resting energy expenditure was approximately 3 500 kcal/day, corresponding to 1.5 times the expected basal metabolic rate for a 120 kg adult male [20].

## 4 Discussion

Both cases illustrate the life-threatening complications associated with DNP consumption. In Case 1, cardiac arrest occurred rapidly after a large ingestion of DNP. In Case 2, DNP was ingested for “therapeutic” purposes in a much smaller total amount, and the patient ultimately survived. However, these differences in patient history and outcome are not reflected in the measured blood DNP concentrations. In Case 1, the patient died 4 h after ingesting 5 g of DNP and had a post-mortem concentration of 8.4 mg/L. In Case 2, the concentration was 28 mg/L, measured 14 h after the last 200 mg dose and after a total dose of only 1.4 g ingested over several days. We believe this discrepancy is caused by post-mortem redistribution of DNP, a phenomenon in which the compound diffuses from blood into tissues after death [21].

The occurrence of post-mortem redistribution of DNP is supported by a small number of case reports where both ante- and post-mortem blood levels were available [22], [23]. Griffiths et al. reported a patient who arrived at hospital 3 h after an acute ingestion of 8 g of DNP and died within the hour [22]. Blood DNP concentration on hospital arrival was 114 mg/L, almost an order of magnitude higher than the 14 mg/L later found in blood obtained at autopsy four days after death. Kopec et al. described a smaller drop, from 120 mg/L in life to 98 mg/L at autopsy 10 h after death in an acute ingestion of 3 g of DNP [23]. These observations suggest that post-mortem plasma-protein degradation and pH shifts promote the redistribution of DNP from blood to tissues. At physiologic pH, DNP is predominantly albumin-bound, and its free fraction mainly exists in the ionized form, favoring intravascular retention. Death, however, creates conditions that increase unbound and protonated DNP, thereby increasing tissue uptake [21], Meyer et al., 2022, [25]. Given that in Case 1, sampling for DNP analysis was undertaken at autopsy five days after death, it is highly likely that the actual concentration present in the blood at the time of death was much higher than the post-mortem concentration of 8.4 mg/L. Notably, similar physicochemical factors that promote post-mortem redistribution—acidosis and altered protein binding—may also occur in vivo under conditions of overdose, plausibly accelerating DNP uptake into skeletal muscle.

The clinical courses observed in our cases are consistent with such a mechanism. Initially mild symptoms—anxiety, restlessness, dyspnea, and a sensation of heat despite normal measured body temperature—progressed within a short time span to severe agitation, hyperthermia, and hyperkalemia, consistent with previous reports of severe DNP toxicity [22], [23], [26], [27], [28], [29], [30], [31], [32], [33], [34]. Rapid deterioration was accompanied by a marked rise in end-tidal and arterial pCO₂ after intubation, reflecting an abrupt ventilatory–metabolic mismatch. In Case 1, EtCO₂ reached 17 kPa and aB-pCO₂ 14 kPa soon after intubation; in Case 2, the corresponding values peaked at 11.7 kPa and 9.7 kPa. Similar extreme elevations were also reported in two fatal cases by McGillis et al. and Newport et al. [31], [34]. In Case 2, the calculated MV_(required)_ at the post-intubation pCO₂ peak was almost 45 L/min, a level virtually unattainable with conventional mechanical ventilation (Fig. 2, Fig. 3) [19].

We propose that the increased CO₂ production from toxic uncoupling initiates a self-amplifying pathophysiological process at the core of DNP toxicity. According to this hypothesis, an elevated CO₂ production causes a local respiratory acidosis, lowering pH in the microenvironment of mitochondria-rich skeletal muscle. This local acidosis (i) reduces albumin binding of DNP, raising the free fraction of the toxin [35], [36], and (ii) shifts the DNP dissociation equilibrium toward its protonated (undissociated), lipid-soluble form, facilitating cellular and mitochondrial uptake. In parallel, (iii) elevated tissue temperature accelerates enzymatic activity and metabolic rate, further amplifying (i) and (ii). Together, these effects reinforce mitochondrial DNP uptake, intensifying uncoupling and driving a positive feedback loop of CO₂ production, acidosis, and heat generation (Fig. 1). Under certain circumstances—particularly overdose—this loop may escalate into *runaway uncoupling*, rapidly leading to systemic acidosis, heat shock, and fatal metabolic collapse.

Experimental studies in mice provide some support for the phenomenon of runaway uncoupling. At sublethal doses, DNP tissue concentrations show no preferential uptake in skeletal muscle compared with liver or cerebral cortex while at higher doses muscle uptake increases disproportionately [37]. In lethal exposures animals died with immediate rigor mortis but without evidence of uncoupling in brain or liver mitochondria—suggesting a self-reinforcing cycle that renders muscle the exclusive lethal target Meyer et al., 2022, [38]. Such feedback may not be unique to DNP. In salicylate poisoning (a weaker uncoupler), acidosis and/or the initiation of mechanical ventilation are recognized harbingers of life-threatening CNS toxicity [39].

In Case 1, cardiac arrest was followed by severe hyperkalemia, peri-mortem rigidity, and a rise in body temperature to 40 °C during resuscitation. Hyperkalemia and rigidity are striking correlates of catastrophic ATP depletion in skeletal muscle, consistent with the proposed feedback mechanism culminating in irreversible *runaway uncoupling*. By contrast, the patient in Case 2 never progressed to critical ATP depletion, despite sharp rises in potassium and temperature around intubation. High-dose glucose supplementation and extracorporeal potassium removal were essential supportive measures, while sedation/relaxation, active cooling and increasing the minute ventilation to maximize CO_2_ elimination may have helped interrupt the feedback loop before the point of no return. Dantrolene, which was administered during this period, may also have contributed modestly by lowering background metabolic demand. However, because it does not address the primary pathology of mitochondrial uncoupling, its therapeutic value in DNP toxicity is likely limited [32], [40]. Interventions aimed at reducing the self-reinforcing feedback of DNP toxicity are summarized in Table 1.

**Table 1:** Supportive strategies to minimize the feedback process leading to runaway uncoupling in severe DNP poisoning.

**Domain**	**Potential strategy**	**Rationale / comments**
Ventilation	If intubated, use high MV (20–25 L/min) and avoid apnea during intubation	Match excessive CO₂ production; prevent systemic hypercapnia and acidosis
Temperature control	Rapid active cooling (ice packs, ice bath, intravascular catheters, CRRT)	Counteract hyperthermia and heat-driven metabolic acceleration
Muscle activity	Neuromuscular blockade (e.g., rocuronium)	Reduce muscle work and secondary heat generation
Metabolic support	High-dose glucose infusion	Maintain euglycemia in the face of increased metabolic demand
Electrolytes	Continuous K⁺ monitoring; removal via CRRT/dialysis;	Prevent arrhythmia and reduce metabolic stress; insulin likely not effective in ATP-depletion; β₂-agonists should be avoided
Extracorporeal	CRRT. Plasmapheresis?	CRRT provides supportive removal of heat/potassium; plasmapheresis removes albumin-bound DNP
Pharmacologic	Dantrolene (low priority)	Limited rationale; does not address the primary mechanism of uncoupling

*Abbreviations*: MV, minute ventilation; CRRT, continuous renal replacement therapy.

In Case 1, as in several other fatal DNP overdoses, there was a brief temporal window separating ingestion from the onset of *runaway uncoupling*. In our patient, the interval was only 3.5 h, possibly shortened by a preceding “priming dose” ingested the previous evening. McGillis et al. and others have reported intervals of 10–15 h between ingestion and death in comparable cases [10], [34]. If the patient presents to hospital within this window, there is a theoretical opportunity for extracorporeal toxin removal, although this approach remains unproven. Hemodialysis is likely ineffective in DNP poisoning, as animal studies have shown albumin binding exceeding 90 % even at highly toxic blood levels [25]. Such extensive albumin binding suggests that plasmapheresis, rather than dialysis, may offer a more rational means of DNP removal. Our platelet respirometry experiments support this interpretation: plasma obtained on day 1 reproduced the same degree of uncoupling when incubated with platelets collected on day 8, indicating that circulating DNP was a key driver of toxicity. The rationale for plasmapheresis is further supported by recent pharmacokinetic studies of DNP in mice showing that plasma concentrations exceed tissue levels by roughly an order of magnitude [24]. Given that gastric absorption appears to be rapid, we propose that the several-hour delay between ingestion and collapse represents the time required for the self-reinforcing feedback mechanism outlined above to gain momentum [41]. As argued above, once the runaway stage is reached, the process is likely irreversible. Thus, attempting extracorporeal toxin removal during the preceding window, though unproven, may represent the only viable treatment option in overdose.

Finally, we examined DNP persistence by comparing blood concentrations with functional indicators of mitochondrial uncoupling. In Case 2, blood DNP declined with an apparent half-life of 16.7 h, yet several independent measures indicated a markedly longer functional persistence. Platelet respirometry suggested a decline in uncoupling with a half-life of approximately 4.9 days. Although platelet uncoupling may have been influenced by normal platelet turnover, the measured decay correlated well with the clinical course (Fig. 4).

Functional uncoupling, as assessed through ventilatory-demand data, exhibited two phases: an initial decline in MV_(required)_ between 2 and 32 h after intubation, followed by a plateau of persistently elevated MV_(required)_ throughout the remaining seven days (Fig. 3). We speculate that the initial decline reflected reduced metabolic demand from sedation, active cooling, and attenuation of stress responses, rather than rapid elimination of DNP from muscle tissue. Ventilator-derived calorimetry on day 6 also indicated elevated metabolic rate, supporting significant continued mitochondrial uncoupling. These observations are consistent with the protracted recovery described in other survivors of DNP poisoning and with animal studies demonstrating a prolonged terminal elimination phase Meyer et al., 2022, [42], [43].

Taken together, our observations demonstrate that defining a safe dosing range for DNP is impossible. The combination of tissue persistence and a self-reinforcing feedback loop in skeletal muscle implies that even apparently moderate exposures may escalate into uncontrollable toxicity. Several contingent factors may further amplify this risk. Co-ingestion of stimulants or doping agents such as clenbuterol, underlying metabolic disorders such as hyperthyroidism, and physical exertion can all accelerate metabolism thereby enhancing mitochondrial uptake through feedback [44]. High ambient temperature is another potent amplifier and has been shown to markedly increase DNP toxicity in rodents [45]. Notably, the illness in Case 2 occurred during a record-breaking regional heat wave, underscoring the role of environmental heat as a clinical risk factor. Historically, fatalities among British agricultural workers exposed to the DNP-like pesticide dinitro-ortho-cresol (DNOC) occurred almost exclusively on hot summer days [46]. These synergistic factors render DNP toxicity inherently unpredictable and reaffirm the FDA’s conclusion reached nearly a century ago: DNP is not fit for human consumption.

## CRediT authorship contribution statement

**Wilhelm Wallquist:** Writing – review & editing, Writing – original draft, Supervision, Data curation, Conceptualization. **Märta Leffler:** Data curation, Conceptualization. **Tine Sommer:** Data curation, Conceptualization. **Erik Lindeman:** Writing – review & editing, Writing – original draft, Conceptualization. **Fredrik Sjövall:** Writing – review & editing, Methodology, Data curation, Conceptualization. **Eskil Elmér:** Writing – review & editing, Methodology, Data curation, Conceptualization. **Shusuke Sekine:** Methodology, Data curation.

## Consent for publication

Written informed consent for publication was obtained from the surviving patient (Case 2). For the deceased patient (Case 1), consent from next of kin was not required as all identifying details have been removed and the case is presented anonymously.

## Ethics statement

Formal ethical approval was not required for this retrospective case report series, which is based on anonymized clinical and post-mortem data.

## Declaration of Generative AI and AI-assisted technologies in the writing process

During the preparation of this work the authors used ChatGPT, OpenAI, GPT-5 solely for grammar, style, and formatting improvements. After using this tool/service, the authors reviewed and edited the content as needed and take(s) full responsibility for the content of the published article.

## Funding

Except for salary funding for S.S. from the Scandinavia Japan Sasakawa Foundation (grants GA22-JPN-0071 and GA24-JPN-0122), this research did not receive any specific grants from funding agencies in the public, commercial, or not-for-profit sectors.

## Declaration of Competing Interest

The authors declare that they have no known competing financial interests or personal relationships that could have appeared to influence the work reported in this paper.

## Data Availability

Data will be made available on request.

## References

[bib1] 1Dinitrophenol: toxic weight loss. J Pharm Technol. 1987;3(3):109–12.

[2] Perkins R.G. (1919). A study of the munitions intoxications in France. Public Health Rep..

[3] Cutting W.C., Mehrtens H.G., Tainter M.L. (1933). Actions and uses of dinitrophenol. J. Am. Med Assoc..

[4] Colman E. (2007). Dinitrophenol and obesity: an early twentieth-century regulatory dilemma. Regul. Toxicol. Pharm..

[5] Tainter M.L., Cutting W.C., Stockton A.B. (1934). Use of dinitrophenol in nutritional disorders. Am. J. Public Health.

[6] Horner W.D. (1941). A study of dinitrophenol and its relation to cataract formation. Trans. Am. Ophthalmol. Soc..

[7] Kurt T.L., Anderson R., Petty C., Bost R., Reed G., Holland J. (1986). Dinitrophenol in weight loss: the poison center and public health safety. Vet. Hum. Toxicol..

[8] Tainter M.L. (1934). A case of fatal dinitrophenol poisoning. J. Am. Med Assoc..

[9] Poole F.E., Haining R.B. (1934). Sudden death from dinitrophenol poisoning. J. Am. Med Assoc..

[10] Grundlingh J., Dargan P.I., El-Zanfaly M., Wood D.M. (2011). 2,4-Dinitrophenol (DNP): a weight-loss agent with significant acute toxicity and risk of death. J. Med Toxicol..

[11] Loomis W.F., Lipmann F. (1948). Reversible inhibition of the coupling between phosphorylation and oxidation. J. Biol. Chem..

[bib12] 12Agency for Toxic Substances and Disease Registry (US). Toxicological profile for dinitrophenols. Atlanta (GA): ATSDR; 2021.

[13] Kotova E.A., Antonenko Y.N. (2022). Fifty years of research on protonophores: mitochondrial uncoupling as a basis for therapeutic action. Acta Nat..

[14] Potts A.J., Bowman N.J., Seger D.L., Thomas S.H.L. (2021). Toxicoepidemiology and predictors of death in 2,4-dinitrophenol (DNP) toxicity. Clin. Toxicol. (Philos. ).

[15] Gziut T., Thomas S.H.L. (2022). International trends in systemic human exposures to 2,4-dinitrophenol reported to poisons centres. Clin. Toxicol. (Philos. ).

[16] Hultén P., Lindeman E. (2025). 2,4-Dinitrophenol poisonings in Sweden 2010–2024: still here, still deadly. Clin. Toxicol. (Philos. ).

[17] Sjövall F., Ehinger J.K.H., Marelsson S.E., Morota S., Åsander Frostner E., Uchino H. (2013). Mitochondrial respiration in human viable platelets: methodology and influence of gender, age and storage. Mitochondrion.

[18] Wexler H.R., Lok P. (1981). A simple formula for adjusting arterial carbon dioxide tension. Can. Anaesth. Soc. J..

[19] Stapel S.N., de Grooth H.J.S., Alimohamad H., Elbers P.W.G., Girbes A.R.J., Weijs P.J.M. (2015). Ventilator-derived carbon dioxide production to assess energy expenditure in critically ill patients: proof of concept. Crit. Care.

[20] Roza A.M., Shizgal H.M. (1984). The Harris–Benedict equation reevaluated: resting energy requirements and the body cell mass. Am. J. Clin. Nutr..

[21] Ferner R.E. (2008). Post-mortem clinical pharmacology. Br. J. Clin. Pharm..

[22] Griffiths A., Milne N., Ong B., Watkins T. (2021). 2,4-Dinitrophenol overdose: everything old is new again. J. Forensic Leg. Med.

[23] Kopec K.T., Freiermuth C., Maynard S., Beuhler M. (2018). Dinitrophenol (DNP) fatality associated with a falsely elevated salicylate level: a case report with verification of laboratory cross-reactivity. J. Med Toxicol..

[bib24] Meyer L.F., Rajadhyaksha P.M., Shah D.K. (2022). Physiologically based pharmacokinetic model for 2,4-dinitrophenol. J. Pharmacokinet. Pharmacodyn..

[25] Mudge G.H., Taggart J.V. (1950). Effect of 2,4-dinitrophenol on renal transport mechanisms in the dog. Am. J. Physiol..

[26] Tewari A., Ali T., O’Donnell J., Butt M.S. (2009). Weight loss and 2,4-dinitrophenol poisoning. Br. J. Anaesth..

[27] Personne M., Ekström M., Iveroth M. (2014). 2,4-Dinitrophenol: a lethal weight-reducing agent. Lakartidningen.

[28] Holborow A., Purnell R.M., Wong J.F. (2016). Beware the yellow slimming pill: fatal 2,4-dinitrophenol overdose. BMJ Case Rep..

[29] Hermetet C., Jourdan M., Baert A., Gheddar L., Ameline A., Kintz P. (2024). Fatal long-term intoxication by 2,4-dinitrophenol and anabolic steroids in a young bodybuilder with muscle dysmorphia. Front Public Health.

[30] Zack F., Blaas V., Goos C., Rentsch D., Büttner A. (2016). Death within 44 days of 2,4-dinitrophenol intake. Int J. Leg. Med.

[31] Newport M.D., Said T. (2020). Fatal poisoning with 2,4-dinitrophenol: learning via case study. J. Paramed. Pr..

[32] Van Schoor J., Khanderia E., Thorniley A. (2018). Dantrolene is not the answer to 2,4-dinitrophenol poisoning: more heated debate. BMJ Case Rep..

[33] Larsen L.G., Halberg L., Bakke S.A. (2015). Fatal course following poisoning with an illegal weight-reducing agent. Ugeskr. Laege.

[34] McGillis E.S., Olives T.D., Love S.A., Cole J.B. (2020). A young man with accelerated hyperthermia, hypercapnia, and profound muscle rigidity after ingestion of a weight-loss agent. Ann. Am. Thorac. Soc..

[35] Kragh-Hansen U., Chuang V.T.G., Otagiri M. (2002). Practical aspects of the ligand-binding and enzymatic properties of human serum albumin. Biol. Pharm. Bull..

[36] van der Giesen W.F., Wilting J. (1983). Consequences of the N–B transition of albumin for the binding of warfarin in human serum. Biochem Pharm..

[37] Geisler J.G. (2019). 2,4-Dinitrophenol as medicine. Cells.

[38] Ilivicky J., Casida J.E. (1969). Uncoupling action of 2,4-dinitrophenols, 2-trifluoromethylbenzimidazoles and certain other pesticide chemicals upon mitochondria from different sources and its relation to toxicity. Biochem Pharm..

[39] Stolbach A.I., Hoffman R.S., Nelson L.S. (2008). Mechanical ventilation was associated with acidemia in a case series of salicylate-poisoned patients. Acad. Emerg. Med.

[40] Kopec K.T., Kim T., Mowry J., Aks S., Kao L. (2019). Role of dantrolene in dinitrophenol (DNP) overdose: a continuing question?. Am. J. Emerg. Med.

[41] Sousa D., Carmo H., Roque Bravo R., Carvalho F., Bastos M.L., Guedes de Pinho P. (2020). Diet aid or aid to die: an update on 2,4-dinitrophenol (2,4-DNP) use as a weight-loss product. Arch. Toxicol..

[42] Barker K., Seger D., Kumar S. (2006). Comment on “pediatric fatality following ingestion of dinitrophenol: postmortem identification of a ‘dietary supplement’. Clin. Toxicol. (Philos. ).

[43] Van Veenendaal A., Baten A., Pickkers P. (2010). Weight loss and 2,4-dinitrophenol poisoning. Br. J. Anaesth..

[44] Dufayet L., Gorgiard C., Vayssette F., Barbet J., Hoizey G., Ludes B. (2020). Death of an apprentice bodybuilder following 2,4-dinitrophenol and clenbuterol intake. Int J. Leg. Med.

[45] Harvey D.G. (1959). On the metabolism of some aromatic nitro compounds by different species of animal: part III. the toxicity of the dinitrophenols, with a note on the effects of high environmental temperatures. J. Pharm. Pharm..

[46] Bidstrup P.L., Payne D.J.H. (1951). Poisoning by dinitro-ortho-cresol. BMJ.

